# Clinicopathologic and molecular landscape of villitis of unknown etiology: insights from a large‐scale case–control study in PR China

**DOI:** 10.1002/2056-4538.70084

**Published:** 2026-03-16

**Authors:** Meiling Wang, Xiaorong Sun, Huayan Ren, Fengchun Gao, Xiangyu Sun, Chang Lu, Yanxue Yin, Juan Li, Chengquan Zhao

**Affiliations:** ^1^ Department of Pathology Jinan Maternity and Child Care Hospital Jinan PR China; ^2^ Department of Pathology The First Affiliated Hospital of Zhengzhou University Zhengzhou PR China; ^3^ Department of Obstetrics Jinan Maternity and Child Care Hospital Jinan PR China; ^4^ Department of Information Jinan Maternal and Child Care Hospital Jinan PR China; ^5^ Department of Pathology Magee‐Womens Hospital, University of Pittsburgh Medical Center Pittsburgh PA USA

**Keywords:** villitis of unknown etiology, placenta, hypertensive disorders of pregnancy, fetal vascular malperfusion, interferon signaling, GBP5

## Abstract

Villitis of unknown etiology (VUE) is a chronic placental inflammatory lesion characterized by lymphohistiocytic infiltration and destruction of villous architecture in the absence of infection. Although VUE is well recognized for its association with fetal growth restriction and adverse pregnancy outcomes, its clinicopathologic correlates and molecular basis remain poorly understood. VUE cases were identified from the pathology database of Jinan Maternal and Child Health Hospital (2020–2023) and classified as high‐grade or low‐grade according to the Amsterdam criteria. Clinical data, maternal complications, and neonatal outcomes were collected from electronic medical records. Multivariable logistic regression was used to determine independent risk factors. RNA sequencing was performed on 28 placental samples (24 VUE and 4 controls) to identify differentially expressed genes and pathways. A total of 970 cases (381 high‐grade and 589 low‐grade) and 980 controls were included. VUE prevalence was 5.8%. Hypertensive disorders of pregnancy (HDP) were independently associated with VUE occurrence (odds ratio 1.67, 95% CI 1.28–2.19, *p* < 0.001). VUE placentas frequently exhibited chronic intervillositis, chronic deciduitis, and avascular villi, whereas maternal vascular malperfusion showed no significant difference from controls. High‐grade VUE was significantly associated with low‐birth‐weight, small‐for‐gestational‐age infants, and increased neonatal intensive care unit admissions, indicating a severity‐dependent impact on neonatal outcomes. Transcriptomic profiling revealed robust upregulation of interferon‐inducible, cytotoxic, and chemokine genes – most notably *GBP5*, *CXCL9*, and *CXCL10* – with enrichment of interferon‐γ, IL‐6/JAK/STAT3, TNF‐α/NF‐κB, and antigen presentation pathways. VUE, particularly its high‐grade form, is a significant placental lesion associated with HDP, adverse neonatal outcomes, and avascular villi. Its distinct interferon‐rich molecular profile, consistent with a maternal anti‐fetal T‐cell–mediated process, underscores its clinical and biological importance. GBP5 emerges as a potential biomarker of interferon‐driven inflammation, providing new mechanistic insight and diagnostic relevance for placental immune injury.

## Introduction

Villitis of unknown etiology (VUE) is a chronic inflammatory lesion of the placenta characterized by lymphohistiocytic infiltration of villous stroma and destruction of villous architecture in the absence of identifiable infectious agents [1, 2]. According to the Amsterdam Placental Workshop Group consensus, VUE can be further classified by severity as low‐grade (LG) or high‐grade (HG), and by distribution into distal, proximal, or basal patterns [3, 4]. VUE occurs in approximately 5–15% of term placentas and is increasingly recognized as an important cause of fetal growth restriction, recurrent pregnancy loss, and stillbirth [1, 2, 5, 6]. Despite its clinical relevance, the precise pathogenesis of VUE remains elusive, representing both a diagnostic and mechanistic challenge in perinatal pathology [2].

Previous histopathologic studies have suggested that VUE represents an aberrant maternal immune response directed against the semi‐allogeneic fetus, sharing immunophenotypic features with allograft rejection [2, 7]. Paternally derived antigens, particularly HLA‐C, can breach maternal–fetal tolerance by activating maternal cytotoxic T cells [8]. Concomitantly, defective regulatory T‐cell function, a skewed Th1/Th17 milieu, and danger‐associated molecular patterns that trigger NLRP3 inflammasome activation amplify sterile inflammation within the villous stroma [9]. Immunohistochemical and molecular analyses have demonstrated abundant maternal CD8^+^ T cells and macrophages within affected villi, accompanied by upregulation of interferon‐induced and cytotoxic/chemokine mediators (e.g., CXCL9/CXCL10) [10, 11]. Alternatively, a subset of cases exhibits transcriptomic and morphologic features resembling antiviral immune activation, suggesting that VUE may encompass heterogeneous mechanisms, including alloimmune and antiviral responses [2, 10, 12]. However, most prior studies were limited by small sample sizes or focused exclusively on morphologic or immunohistochemical findings, with limited integration of molecular profiling and clinicopathologic correlation.

In clinical practice, VUE is frequently associated with adverse perinatal outcomes, including fetal growth restriction, low birth weight (LBW), and preterm delivery, and the lesion shows a tendency to recur in subsequent pregnancies [2, 5, 13]. VUE also often coexists with other placental lesions such as fetal vascular malperfusion (FVM), which themselves are linked to impaired perfusion and adverse neonatal outcomes [14, 15]. Nevertheless, the relationship between the severity of villitis, concomitant lesions (e.g., FVM), and neonatal outcomes remains incompletely defined, and the molecular pathways underlying VUE – and their contribution to disease heterogeneity – have not been comprehensively elucidated [2, 14].

To address these knowledge gaps, we conducted a large‐scale clinicopathologic study of 970 cases of VUE and 980 controls to characterize maternal risk factors, associated placental lesions, and neonatal outcomes. In addition, RNA sequencing of formalin‐fixed paraffin‐embedded (FFPE) placentas was performed to delineate the transcriptional landscape of VUE and identify key molecular pathways involved in its pathogenesis. Our findings provide new insights into the clinical correlations and immune mechanisms of VUE, offering evidence‐based guidance for improved prenatal risk assessment, individualized management, and neonatal care.

## Materials and methods

### Ethics statement

All procedures were performed in accordance with relevant laws and institutional guidelines and were approved by the Institutional Review Committee of the Jinan Maternity and Child Care Hospital (JMCH) (approval number KYR24‐067, 8 May 2024), a nonprofit, academic, and tertiary‐level Class A maternity and childcare hospital in PR China.

### Case selection

During this period, 48,357 deliveries occurred at JMCH, and 16,945 placentas were submitted for pathology examination, including 16,267 singleton pregnancies. The pathology database was queried for cases diagnosed as VUE between January 2020 and December 2023. Placental pathology examinations were recommended by obstetricians whenever standard clinical indications were present, as previously described [16], and were conducted with maternal consent. For this study purpose, all placental slides of the VUE cases were reviewed by a gynecologic pathologist (JL) to confirm the diagnosis according to the Amsterdam criteria [16]. After excluding cases with chromosomal aneuploidy, major genetic disorders, or multiple gestations, the cases with a confirmed diagnosis of VUE were included for this study. The control group consisted of placentas submitted for routine histopathologic evaluation during the same study period, with definitive absence of VUE. Maternal and neonatal clinical data were recorded from medical charts.

### Gross and histological examination of placentas

The placentas were processed routinely in the Department of Pathology. Upon receipt, the specimens were placed in 10% neutral buffered formalin, fixed for 24 h, weighed, and measured. Subsequently, the tissues were processed using an automated tissue processor with a standard 14‐h protocol, embedded in conventional paraffin, and sectioned at a thickness of 5 μm. The gross examination and histological sampling were performed according to the protocol of the Amsterdam Placental Workshop Group Consensus Statement [17].

VUE was diagnosed and graded according to the Amsterdam criteria [17]. HG VUE was defined as inflammation involving ≥10 contiguous villi in at least one focus or as patchy/diffuse inflammation affecting multiple separate foci, whereas LG VUE was defined as inflammation involving <10 contiguous villi per focus, with more than one focus required for diagnosis.

### Immunohistochemistry

FFPE placental tissue sections were routinely dewaxed and rehydrated. Antigen retrieval was performed by heating sections in EDTA buffer (pH 9.0) in a pressure cooker, maintaining a boil for 2 min, followed by cooling to room temperature. Sections were then incubated overnight at 4 °C in a humidified chamber with the following primary antibodies: GBP5 (Proteintech, Cat. No. 13220‐1‐AP, Wuhan, PR China), CD68 (ZSGB‐BIO, Cat. No. ZM‐0060), CD8 (ZSGB‐BIO, Cat. No. ZA‐0508), or CD34 (ZSGB‐BIO, Cat. No. ZA‐0550, Beijing, PR China). After rewarming and thorough washing with PBS, the sections were incubated with an appropriate HRP‐conjugated secondary antibody polymer for 40 min at 37 °C. The immunoreaction was visualized using freshly prepared DAB (3,3′‐diaminobenzidine) chromogen.

### RNA sequencing (RNA‐seq) and bioinformatics analysis

For transcriptional profiling, cases demonstrating HG VUE patterns were prioritized to ensure the capture of representative molecular alterations associated with severe inflammation. RNA‐seq and initial bioinformatics processing of FFPE placental samples were performed by CNKINGBIO Biotech Inc. (Beijing, PR China). Total RNA was extracted from FFPE tissues after deparaffinization, and RNA quality was assessed using DV200 values, as RIN is not suitable for FFPE‐derived RNA. Samples with acceptable DV200 >30% were used for library construction. RNA‐seq libraries were generated using an rRNA‐depletion–based whole‐transcriptome protocol optimized for degraded FFPE RNA. Ribosomal RNA was removed, and the remaining RNA fragments were reverse‐transcribed to synthesize first‐ and second‐strand cDNA, followed by end repair, A‐tailing, adapter ligation, and PCR amplification. Sequencing was performed on the Illumina platform to obtain 2 × 150 bp paired‐end reads.

Raw sequencing data were processed by CNKINGBIO using a standard RNA‐seq pipeline. After adapter trimming and quality filtering, clean reads were aligned to the human reference genome (GRCh38) using STAR. Gene‐level counts were quantified using featureCounts. Differential expression analysis was performed using the DESeq2 package in R, with genes meeting the criteria of *p* < 0.05 and |log_2_FC| > 1 considered as differentially expressed. KEGG pathway enrichment and Gene Set Enrichment Analysis (GSEA) were conducted using the clusterProfiler R packages.

### Statistical analysis

All statistical analyses were performed using SPSS version 25.0 (IBM Corp., Armonk, NY, USA). This study included 970 VUE cases and 980 controls. An *a priori* power calculation was conducted for an unmatched case–control study, which indicated that our sample size had 99.8% power (*α* = 0.05, two‐sided) to detect an odds ratio (OR) of 2.0, assuming a 10% exposure rate in the control group. For univariate analyses comparing baseline characteristics among the three groups (control, LG VUE, and HG VUE), one‐way ANOVA or the Kruskal–Wallis test was used for continuous variables, depending on distributional assumptions. Pearson's chi‐square test or Fisher's exact test was used for categorical variables, as appropriate. To identify independent risk factors for VUE while adjusting for potential confounders, a multivariable binary logistic regression model was constructed to estimate adjusted odds ratios (aORs) and 95% confidence intervals (CIs). Covariates that were clinically relevant or those with *p* ≤ 0.05 in univariate analysis were included in the model. A two‐sided *p* < 0.05 was considered statistically significant.

## Results

### Maternal characteristics and associated risk factors for VUE

During the study period, a total of 48,357 deliveries occurred at JMCH, of which 16,945 placentas were submitted for pathological examination, including 16,267 singleton pregnancies. Among these, 970 cases of VUE were identified, including 381 HG and 589 LG cases, yielding an overall VUE prevalence of 5.8% in singleton pregnancies. Another 980 singleton placentas were selected as controls.

Maternal age and gestational age at delivery were comparable among the control, LG VUE, and HG VUE groups (both *p* > 0.05). However, maternal complications such as hypertensive disorders in pregnancy, gestational diabetes mellitus, and a scarred uterus were more common in VUE pregnancies, particularly in the HG group (Table 1). The prevalence of hypertensive disorders increased from 11.8% in the control group to 14.9% in LG VUE and 21.3% in HG VUE (*p* < 0.001). Similarly, the incidence of gestational diabetes mellitus and the history of cesarean delivery were significantly higher in VUE pregnancies (*p* < 0.05).

**Table 1:** Comparison of maternal characteristics, placental histopathologic lesions, and neonatal outcomes among control, low‐grade VUE, and high‐grade VUE groups

	Control (*n* = 980)	VUE		*p*
		Low‐grade (*n* = 589)	High‐grade (*n* = 381)	
Maternal conditions				
Age (years), mean	32.51	32.48	32.55	>0.05
Gestational age (weeks), mean	38.18	38.33	37.94	>0.05
Number of deliveries >1	244 (24.9%)	199 (33.8%)	146 (38.3%)	<0.001
Gestational diabetes mellitus	255 (26.0%)	192 (32.6%)	123 (32.2%)	0.007
Hypertensive disorders of pregnancy	116 (11.8%)	88 (14.9%)	81 (21.3%)	<0.001
Hypothyroidism	123 (12.6%)	66 (11.2%)	60 (15.7%)	0.114
Vaginitis	214 (21.8%)	145 (24.6%)	82 (21.5%)	0.378
Scarred uterus	130 (13.3%)	98 (16.6%)	72 (18.9%)	0.022
Placental lesions				
Chronic inflammation	39 (4.0%)	106 (18.0%)	74 (19.4%)	<0.001
Acute inflammation	59 (6.0%)	31 (5.3%)	27 (7.1%)	0.517
Avascular villi	76 (7.8%)	99 (16.8%)	117 (30.7%)	<0.001
Maternal vascular malperfusion	35 (3.6%)	29 (4.9%)	18 (4.7%)	0.391
Neonatal outcomes				
NICU admission	245 (25.1%)	135 (22.9%)	121 (31.8%)	0.007
Low birth weight	85 (8.7%%)	63 (10.7%)	74 (19.4%)	0.001
Small for gestational age	10 (1.0%)	14 (2.4%)	21 (5.5%)	<0.001
Neonatal hyperbilirubinemia	75 (7.7%)	43 (7.3%)	40 (10.5%)	0.156
Neonatal pneumonia	133 (13.6%)	68 (11.5%)	59 (15.5%)	0.201

The *p* values represent comparisons among the three groups (control, low‐grade VUE, and high‐grade VUE), using chi‐square tests for categorical variables and one‐way ANOVA for continuous variables.

NICU, neonatal intensive care unit; VUE, villitis of unknown etiology.

To further identify independent maternal factors associated with VUE, a multivariable logistic regression analysis was performed (Table 2). Hypertensive disorders in pregnancy remained independently associated with the occurrence of VUE (aOR = 1.67, 95% CI 1.28–2.19, *p* < 0.001), whereas gestational diabetes mellitus, a scarred uterus, and hypothyroidism were not significant after adjustment. These findings indicate that maternal hypertension may be an independent risk factor for the development of VUE.

**Table 2:** Multivariable logistic regression analysis of maternal factors associated with villitis of unknown etiology (VUE)

Exposure	aOR	95% CI (lower)	95% CI (upper)	*p*
Hypertensive disorders in pregnancy	1.671	1.275	2.189	<0.001
Gestational diabetes mellitus	1.190	0.970	1.460	0.095
Scarred uterus	0.949	0.728	1.235	0.695
Hypothyroidism	1.070	0.812	1.410	0.631

Adjusted odds ratios (aORs) and 95% confidence intervals (CIs) were derived from a multivariable logistic regression model including hypertensive disorders in pregnancy, gestational diabetes mellitus, scarred uterus, and hypothyroidism.

### Concomitant placental lesions associated with VUE

**Figure 1:**
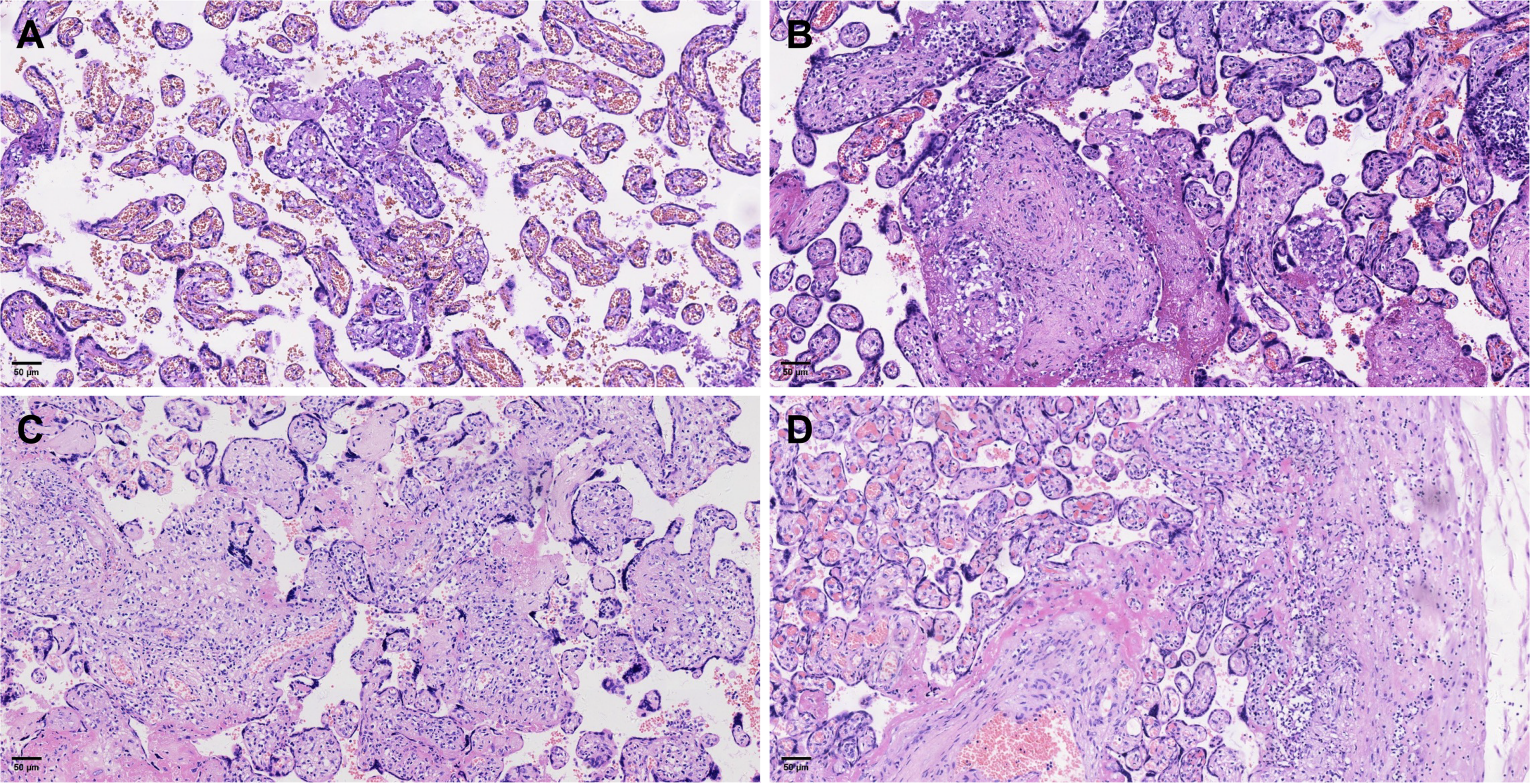
Representative histologic features of VUE and associated placental lesions. (A) Low‐grade VUE characterized by mild lymphohistiocytic inflammation involving scattered distal villi. (B) High‐grade VUE showing dense inflammatory infiltrates with more confluent involvement of villous parenchyma. (C) Concomitant FVM, demonstrated by avascular villi and associated stromal changes. (D) Concomitant chronic inflammatory lesions, including chronic intervillositis and mild deciduitis.

Placental histopathologic examination revealed the presence of distinct concomitant pathological features in VUE compared with control placentas (Table 1). Specifically, we evaluated acute and chronic inflammatory lesions, as well as MVM and FVM. Chronic inflammatory lesions, including chronic intervillositis and chronic deciduitis, were significantly more frequent in VUE placentas than in controls (*p* < 0.001). Similarly, the prevalence of avascular villi increased from 7.8% in controls to 16.8% in LG and 30.7% in HG VUE (*p* < 0.001). In contrast, the frequency of MVM and acute inflammatory changes showed no significant difference among the three groups (*p* > 0.05). These findings indicate that VUE, particularly in its HG form, is frequently accompanied by fetal circulatory impairment and chronic inflammatory processes within the placenta. Representative histologic features corresponding to these findings are shown in Figure 1.

### Adverse neonatal outcomes associated with VUE

Neonatal outcomes differed significantly among the study groups (Table 1). Infants from pregnancies complicated by VUE, particularly those with HG lesions, had significantly lower mean birth weights and higher rates of neonatal intensive care unit (NICU) admission than those from control pregnancies (*p* < 0.05). The incidences of LBW and small‐for‐gestational‐age (SGA) infants were also significantly higher in the HG VUE group than in both the LG VUE and control groups, indicating a severity‐dependent relationship between VUE and adverse perinatal outcomes. In contrast, the frequencies of neonatal hyperbilirubinemia and pneumonia did not differ significantly among the three groups (*p* > 0.05).

### Molecular signatures and pathway analysis of VUE placentas

**Figure 2:**
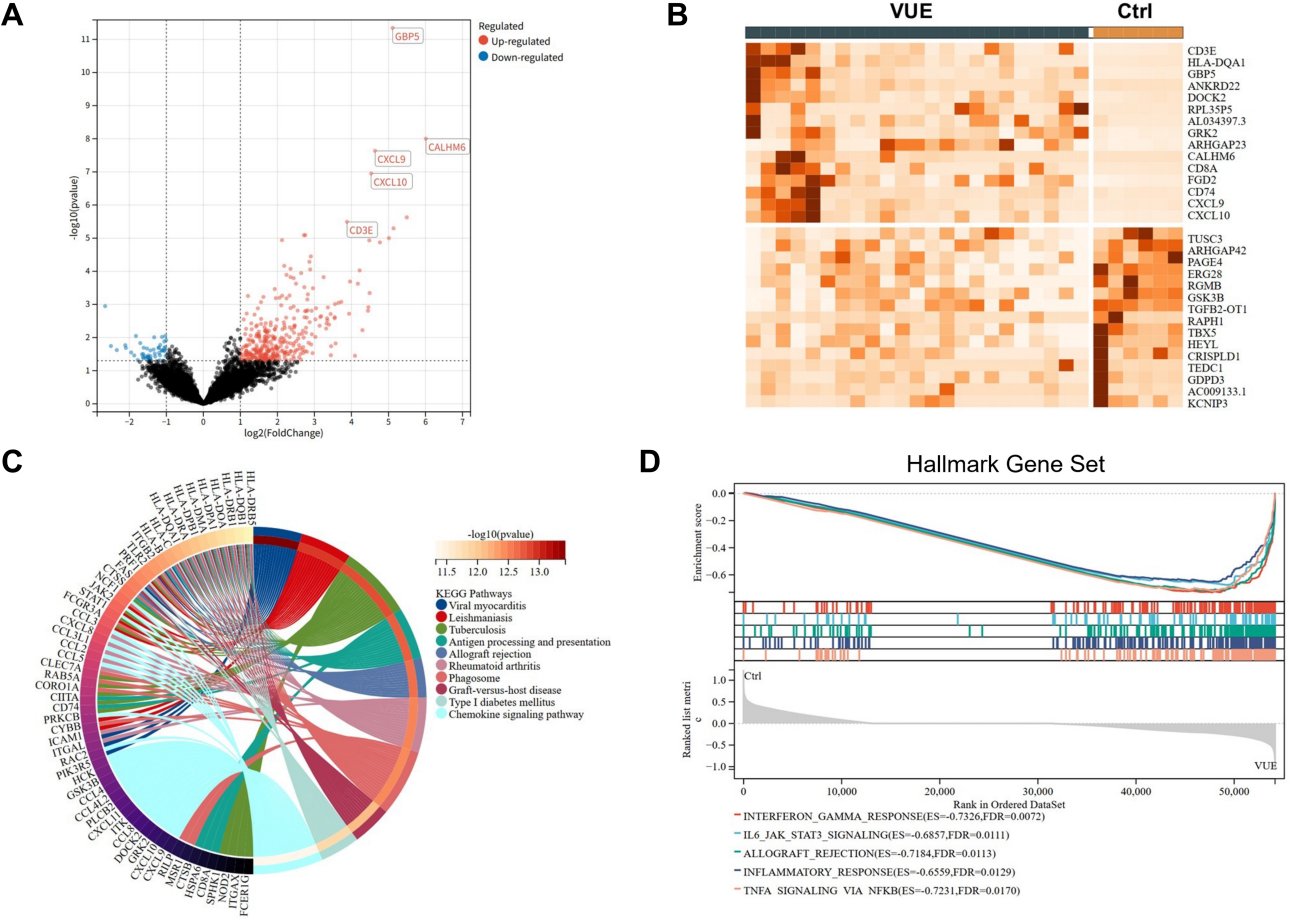
Molecular signatures and pathway enrichment analysis of VUE placentas. (A) Volcano plot showing differentially expressed genes between VUE and control placentas (|log2FC| > 1.5, *p* < 0.05). Representative upregulated genes (*GBP5*, *CALHM6*, *CXCL9*, *CXCL10*, *CD3E*) are highlighted. (B) Heatmap illustrating the expression patterns of immune‐ and interferon‐related differentially expressed genes (*Z*‐score normalized) in VUE versus control placentas. (C) KEGG pathway enrichment analysis of differentially expressed genes. The circle plot depicts the top enriched pathways, including viral myocarditis, allograft rejection, chemokine signaling, and antigen processing and presentation pathways. (D) Gene Set Enrichment Analysis (GSEA) demonstrating significant enrichment of interferon‐γ response, IL‐6/JAK/STAT signaling, allograft rejection, inflammatory response, TNF‐α signaling via NF‐κB, and interferon‐α response in VUE placentas.

**Figure 3:**
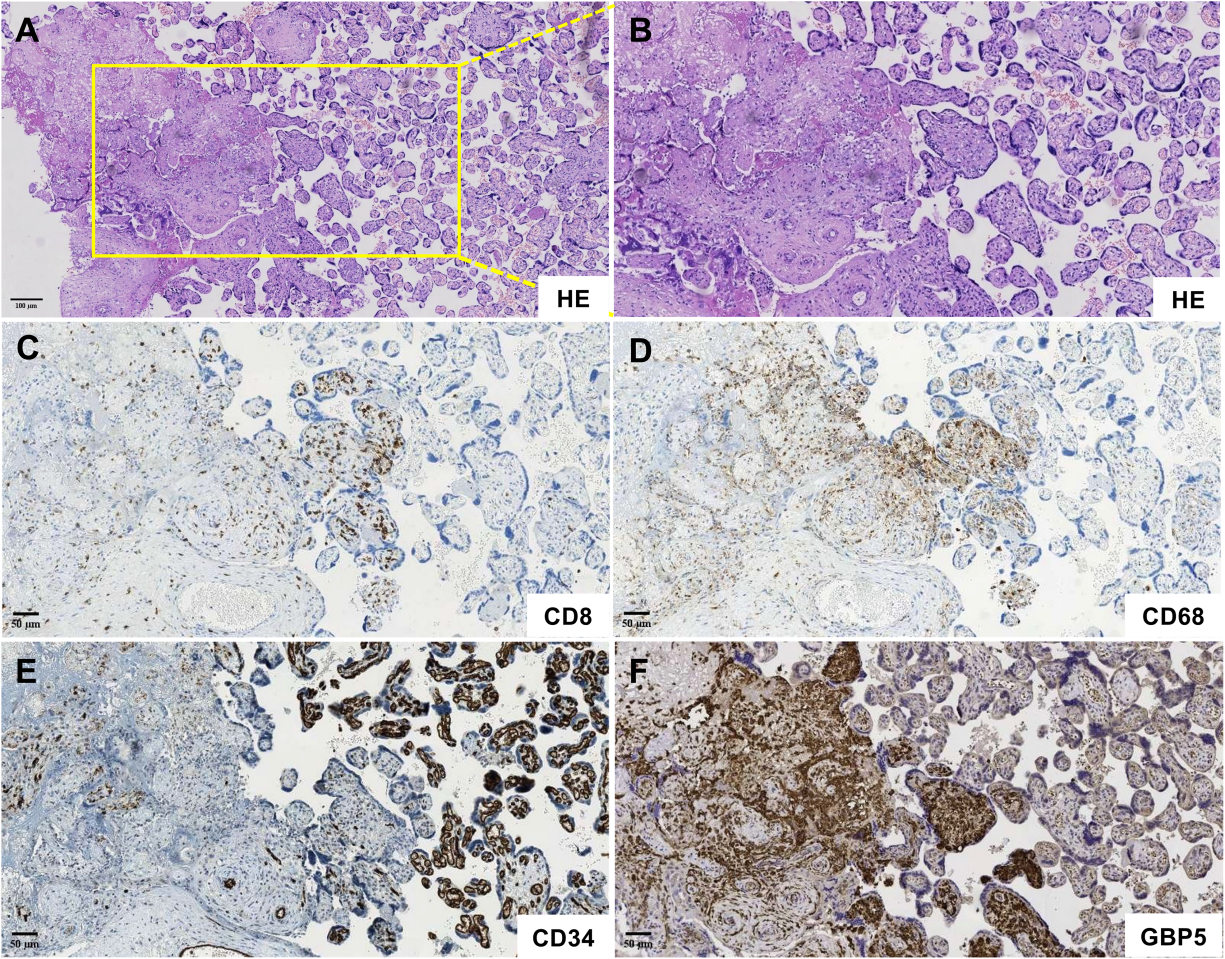
Histopathological and immunohistochemical features of VUE. (A and B) Hematoxylin–eosin (HE) staining of VUE placentas showing dense lymphohistiocytic infiltrates within villous stroma, associated with villous damage, stromal collapse, and patchy necrosis. (C) CD8 immunostaining demonstrating prominent accumulation of CD8^+^ T cells within inflamed villi and around disrupted villous structures. (D) CD68 immunostaining showing extensive infiltration of villous stromal macrophages and intervillous histiocytes. (E) CD34 highlighting reduced and focally attenuated fetal capillaries in affected villi, consistent with localized vascular injury. (F) GBP5 immunohistochemistry revealing strong cytoplasmic expression in villous stromal macrophages and lymphocytes, with additional moderate staining in stromal cells and focal positivity in villous endothelial cells.

To investigate the molecular landscape underlying VUE, RNA sequencing was performed on 28 FFPE placental samples, including 24 HG‐VUE cases and 4 non‐VUE controls. Differential expression analysis identified 338 significantly upregulated and 48 downregulated genes in VUE compared with controls (adjusted *p* < 0.05, |log_2_FC| > 1). Among these, *GBP5* emerged as the most significantly upregulated gene (Figure 2A), together with other inflammation‐related transcripts such as CXCL9 and CXCL10. Given the prominent transcriptional upregulation of GBP5, we next examined its spatial expression in placental tissues from 10 HG‐VUE cases and controls (Figure 3). Immunohistochemistry demonstrated strong cytoplasmic staining of GBP5 in villous stromal macrophages and lymphocytes, with additional moderate expression in stromal cells and focal positivity in villous endothelial cells in VUE (Figure 3F). These findings were concordant with the RNA‐seq results and highlighted GBP5 as a lesion‐specific marker within inflamed villi. These GBP5 expression patterns, spanning macrophages, lymphocytes, stromal cells, and endothelial cells, are consistent with IFN‐γ–induced activation across multiple cellular compartments in VUE placentas.

Histopathological examination further supported these molecular alterations. Hematoxylin–eosin (HE) staining revealed dense lymphohistiocytic infiltrates involving the villous stroma space, accompanied by villous distortion, stromal collapse, and patchy necrosis (Figure 3A, B). CD8 immunostaining confirmed prominent accumulation of cytotoxic T cells (Figure 3C), while CD68 highlighted extensive infiltration of villous macrophages within inflamed regions (Figure 3D). CD34 staining demonstrated reduced and attenuated fetal capillaries in affected villi (Figure 3E), suggesting localized vascular compromise secondary to inflammation.

Heatmap profiling of the top differentially expressed genes in the VUE cohort demonstrated markedly increased expression of immune‐related transcripts – such as *CXCL9* and *CXCL10* – which are associated with leukocyte chemotaxis and activation (Figure 2B). Pathway enrichment analysis revealed that these upregulated genes were predominantly involved in immune system regulation, antigen processing and presentation, cytokine–cytokine receptor interactions, and angiogenesis (Figure 2C). Furthermore, gene set enrichment analysis (GSEA) corroborated these findings, demonstrating enrichment of pro‐inflammatory pathways – including interferon‐γ (IFN‐γ) response, IL‐6/JAK/STAT3 signaling, TNF‐α signaling via NF‐κB, and allograft rejection – in VUE placentas (Figure 2D).

Collectively, the integration of transcriptomic, histopathological, and immunohistochemical evidence indicates that VUE placentas exhibit robust activation of interferon‐mediated immune signaling and T‐cell–driven inflammation, supporting an alloimmune or antiviral mechanism underlying its pathogenesis.

## Discussion

In this large clinicopathologic study integrated with whole transcriptomic profiling, we demonstrate that VUE represents a distinct maternal T‐cell–mediated inflammatory process with characteristic clinical associations, accompanying placental lesions, and severity‐dependent adverse neonatal outcomes. Our findings support an interferon‐driven immune activation model and identify hypertensive disorders of pregnancy (HDP) as an independent maternal risk factor, refining current understanding of VUE pathogenesis and clinical relevance.

After multivariable adjustment, HDP were independently associated with the occurrence of VUE in our cohort. This observation aligns with prior studies linking systemic maternal inflammation, endothelial dysfunction, and placental stress in hypertensive pregnancies [1, 13, 18]. Recent transcriptomic profiling by Horii *et al* showed that preeclamptic placentas frequently harbor concurrent VUE with enriched interferon and antigen‐processing pathways, supporting an inflammation‐driven subtype of HDP [19]. Interestingly, Agrawal *et al* found that placental angiogenic balance markers, including placental growth factor, remained within normal limits in most VUE cases, reinforcing the concept of an immune‐ rather than hypoxia‐driven pathogenesis [20]. Ischemic or oxidative stress within the placenta may release danger‐associated molecular patterns that activate the NLRP3 inflammasome and lower the threshold for maternal cytotoxic T‐cell responses [21]. However, the nature of this association warrants a more cautious interpretation. The relationship between VUE and maternal hypertension may be bidirectional. While the vascular stress associated with hypertension might facilitate VUE development, it is equally plausible that the intense inflammatory milieu of VUE could contribute to maternal systemic endothelial dysfunction and hypertension. Alternatively, both conditions may stem from a shared defect in maternal‐fetal immune tolerance. Further prospective studies are required to disentangle these intertwined pathways. Although causality cannot be inferred from this retrospective study, our findings suggest that maternal hypertension may predispose to VUE by amplifying maternal–fetal immune dysregulation through inflammasome‐related pathways.

VUE was significantly associated with chronic inflammatory lesions, including chronic intervillositis and chronic deciduitis, as well as avascular villi, which are the main pathological findings of FVM, whereas MVM and acute inflammatory changes did not differ significantly among groups. The prevalence of avascular villi increased stepwise from controls to LG and HG VUE (7.8%, 16.8%, and 30.7%, respectively), indicating a strong link between villous inflammation and fetal circulatory compromise. Similar associations have been described by Tamblyn *et al* and Osborne *et al*, suggesting that VUE preferentially affects the fetal vascular compartment *via* cytokine‐mediated endothelial injury and microthrombus formation [18, 22, 23, 24]. The frequent coexistence of chronic inflammatory lesions underscores the chronic, sterile, adaptive‐immune nature of VUE rather than an acute infectious etiology [1, 18, 25]. Such fetal vascular injury likely represents the mechanistic bridge between villous inflammation and adverse neonatal outcomes. Notably, the high prevalence of avascular villi observed in our HG VUE cases likely represents obliterative fetal vasculopathy secondary to stromal inflammation, rather than primary FVM caused by upstream obstruction. These observations highlight that chronic immune‐mediated villous injury may not only impair fetal circulation but also coexist with other chronic inflammatory pathologies, reflecting a shared maternal immune milieu.

Consistent with previous studies, we found that the histologic severity of VUE correlated strongly with adverse neonatal outcomes [1, 13, 26]. Infants from pregnancies complicated by HG VUE exhibited significantly lower birth weights, higher rates of SGA status, and increased NICU admissions compared with those from LG VUE and controls. These findings align with the recent large‐scale case–control analysis by Rose *et al*, which identified HG VUE as an independent predictor of SGA (p = 0.01) and further highlighted its contribution to neonatal morbidity [27]. This severity‐dependent trend suggests that quantifying villous inflammatory burden carries prognostic value. However, other cohorts noted that lesion topography, rather than grade alone, may influence clinical outcomes, underscoring the heterogeneity of VUE [14, 22]. The frequent coexistence of fetal vascular compromise in HG lesions further supports a mechanistic link between villous inflammation, impaired perfusion, and fetal growth restriction. Incorporating VUE grading and associated vascular lesions into routine placental pathology reports may therefore improve perinatal risk stratification and neonatal management.

RNA sequencing revealed robust upregulation of interferon‐inducible genes and chemokines, notably *GBP5*, *CXCL9*, and *CXCL10*, accompanied by enrichment of interferon‐γ response, IL‐6/JAK/STAT3 signaling, TNF‐α/NF‐κB signaling, antigen presentation, and allograft‐rejection pathways. These results mirror prior immunophenotypic studies demonstrating maternal CD8^+^ T‐cell and macrophage infiltration with upregulation of cytotoxic and chemokine mediators [18]. *GBP5* was the most significantly upregulated gene and demonstrated lesion‐specific expression by immunohistochemistry, suggesting its potential as a biomarker of inflammatory activation. As a guanylate‐binding protein, GBP5 participates in inflammasome priming and antimicrobial defense; its induction in VUE implies a functional link between interferon signaling and inflammasome activation [21]. Additionally, Gabby *et al* demonstrated increased PD‐L1 expression within VUE lesions, potentially reflecting a compensatory checkpoint‐mediated attempt to restore maternal–fetal immune tolerance [28]. Collectively, these transcriptomic findings reinforce a model in which VUE encompasses overlapping alloimmune and antiviral‐like immune response states [12, 18]. To our knowledge, this is the first study to identify *GBP5* upregulation in VUE placentas, providing molecular evidence of convergent interferon and cytotoxic T‐cell pathways in its pathogenesis.

Strengths of this study include its large, well‐characterized cohort, blinded subspecialty placental pathology review based on Amsterdam criteria, and integration of clinical, histologic, and molecular data. Limitations include potential selection bias (as only placentas submitted for pathologic evaluation were analyzed), and the retrospective design. The absence of maternal–fetal HLA typing and T‐cell receptor repertoire analysis precluded direct assessment of alloimmune mechanisms. Despite these limitations, the integration of clinicopathologic and transcriptomic data provides a comprehensive framework for understanding VUE heterogeneity. Future prospective, multicenter studies employing single‐cell or spatial transcriptomics will be essential to map the cellular circuits driving this lesion.

## Conclusions

This study establishes VUE as a predominantly interferon‐rich, T‐cell–driven placental inflammatory lesion associated with fetal vascular injury and adverse neonatal outcomes. The independent association with maternal hypertension suggests shared upstream pathways of placental stress and immune activation. GBP5 protein emerges as a novel molecular marker of interferon‐driven inflammation and a potential diagnostic adjunct. Incorporating VUE grading and accompanying lesions into routine placental reporting may enable more individualized prenatal surveillance and improved neonatal care.

## Author contributions statement

MW and XS collected the data and drafted the manuscript. CL, XY and XS performed the pathological evaluation, reviewed and edited the manuscript. FG, JL and CZ supervised the study and reviewed and edited the manuscript. All authors read and approved the final manuscript and met the authorship requirements.

## Data Availability

The RNA‐sequencing data generated in this study have been deposited in the Genome Sequence Archive in the China National Center for Bioinformation under accession number PRJCA056322. The clinical and pathological datasets generated during and/or analyzed during the current study are available from the corresponding author on reasonable request.
